# Overproduction of β-barrel outer membrane proteins in *Escherichia coli* BL21(DE3) induces hypervesiculation

**DOI:** 10.20517/evcna.2025.27

**Published:** 2025-07-17

**Authors:** Saloni Sahu, Gregory Koningstein, Catalin M. Bunduc, Nicole van der Wel, Joen Luirink, Peter van Ulsen

**Affiliations:** ^1^Molecular Microbiology, AIMMS and A-LIFE, Vrije Universiteit Amsterdam, De Boelelaan 1108, Amsterdam 1081 HZ, The Netherlands.; ^2^Electron Microscopy Centre Amsterdam, Amsterdam University Medical Centre, Amsterdam 1100 DE, The Netherlands.

**Keywords:** Outer membrane vesicles, β-barrel proteins, BL21(DE3), BAM complex

## Abstract

**Aim:** Gram-negative bacteria release outer membrane vesicles (OMVs) that fulfill many functions including survival during stress conditions, delivery of virulence factors, and nutrient acquisition. Additionally, they are increasingly used as an alternative for live bacteria in vaccine development and as a platform for bioengineering. Recently, OMVs have also been applied to express recombinant outer membrane proteins (OMPs) in their natural context as an alternative to the cumbersome reconstitution in liposomes. Here, we use an *Escherichia coli* strain that lacks four major OMPs for selective expression of the β-barrel assembly machinery (BAM) complex and PhoE in OMVs.

**Methods:** OMV production of *Escherichia coli* BL21(DE3) and its omp8 derivative upon overexpression of BAM and PhoE is compared and characterized.

**Results:** We find that overexpression of the BAM complex and PhoE causes a strong hypervesiculation phenotype, and the OMVs produced are intact and appear to recruit the BamA subunit of BAM and PhoE in their correctly folded and assembled conformations.

**Conclusion:** While the exact mechanism of hypervesiculation remains to be elucidated, it contributes to the suitability of the BL21(DE3)omp8 host strain to produce recombinant OMP-enriched OMVs that can be used for various purposes, including structural analysis.

## INTRODUCTION

The outer membrane (OM) of Gram-negative bacteria is an asymmetric bilayer consisting of an inner leaflet of regular phospholipids and an outer leaflet of lipopolysaccharide (LPS)^[1,2]^. Together, they form a barrier toward external agents, including standard-of-care antibiotics. The OM is home to β-barrel type transmembrane proteins that participate in multiple physiological processes including nutrient uptake. In addition, lipoproteins are anchored in the OM with their active part protruding into the periplasm or external space. In *Escherichia coli*, the thin peptidoglycan (PG) network in the periplasm is connected to the OM via the periplasmic domains of OmpA and Braun’s lipoprotein. Together, the connected PG and OM form a mechanical layer that protects cellular integrity and provides osmoresistance^[3]^.

Extracellular vesicles can be formed in Gram-negative bacteria in two ways: by explosive lysis or by blebbing of the OM. Here we focus on the latter, whereby the OM can expand and form small vesicles (20-200 nm) that bud from the membrane and are released into the extracellular environment^[4,5,6]^. These outer membrane vesicles (OMVs) include lipids, LPS, outer membrane proteins (OMPs), and encapsulated periplasmic content that mimics their parental cellular equivalent, although some proteins may be enriched in or, rather, be excluded from the OMVs. The mechanism of OMV formation is largely unclear but appears well-regulated and specific. Stress conditions that contribute to OMV formation include the accumulation of misfolded proteins in the periplasm and OM, alterations in LPS content and structure, and impaired connection of the OM to the underlying PG layer. In recent years, evidence is accumulating that OMVs play an important role not only in cell stress relief and envelope homeostasis but also in a plethora of other physiological processes such as nutrient acquisition, horizontal gene transfer, and interbacterial communication^[5,7]^. Importantly, OMVs can neutralize antibiotics and phages. Further links to pathogenesis include the induction of biofilm formation, modulation of host immune responses, and serving as a secretion and delivery vehicle for toxins^[5,7]^.

The versatility of OMVs is exploited in biotechnology and medical applications. For instance, OMVs are used as nanocarriers for enzymes that can be either encapsulated to improve stability or exposed at the surface for use in bioremediation and enzymatic cascade reactions^[5,7]^. Currently, there is a great deal of interest in OMVs as a versatile platform for cancer immunotherapy, and antigen delivery in vaccine applications^[8,9,10]^. In the latter modality, they form an attractive, safe (non-replicating) alternative for live attenuated vaccines with which they share a strong intrinsic adjuvant activity. Recent developments involve bioengineered OMVs that are decorated with recombinant antigens, which are either genetically incorporated into surface-exposed carrier proteins, or enzymatically or chemically coupled to such carrier proteins^[5,9,10]^.

To display recombinant antigens at the surface of *Salmonella* typhimurium OMVs, we have developed a “display” platform using a non-secreted variant of the autotransporter *E. coli* hemoglobin protease (Hbp), in which surface-exposed parts were replaced by antigens^[11,12]^. Hbp is an autotransporter that follows a two-step secretion/display mechanism^[9,13]^. Transport of Hbp across the OM is mediated by its C-terminal β-barrel domain, which also involves the β-barrel assembly machinery (BAM), a general system required for the insertion and folding of most β-barrel type OMPs^[1,13]^. To increase the display efficiency of Hbp fusions on the OMVs, we have co-overexpressed the BAM^[14,15]^.

During these studies, we noticed that overexpression of BAM significantly increased the number of OMVs formed. Here, we have investigated this hypervesiculation phenotype in more detail. We find that the effect is particularly apparent in the *E.coli* BL21(DE3)omp8 strain that lacks the major OMPs OmpA, OmpF, OmpC, and LamB^[16]^. Overexpression of the OMP PhoE also caused hypervesiculation, indicating that the effect is not specific for BAM. Further characterization of the OMVs indicated that they are fully intact and the overproduced OMPs are recruited in a properly folded and assembled conformation in the correct orientation. Therefore, overproduction of β-barrel type OMPs in BL21(DE3)omp8 appears to be a promising generic strategy to overproduce OMPs in their native context in the absence of major endogenous OMPs. Potential applications of the generated OMVs are in biotechnology and vaccine development, but also in the structural analysis of OMPs, predominantly by cryotomography, for which the nanosized OMVs are specifically suitable.

## METHODS

### Bacterial strains, plasmids, and culture conditions

Bacterial cells were grown in lysogeny broth (LB) composed of yeast extract (5 g/L; Oxoid), tryptone (10 g/L; Oxoid), and NaCl (10 g/L). The antibiotics ampicillin (Amp) and chloramphenicol (Cam) were added at 100 and 30 µg/mL, respectively, where appropriate. Primary cultures were grown in 5 mL LB overnight and then used to inoculate secondary cultures with an initial optical density at 600 nm (OD_600_) of ~0.05. The cultures were incubated at 37 °C and at 200 rpm. Cells were induced with isopropyl-α-d-thiogalactopyranoside (IPTG) for 2 h, starting at mid-log phase (OD_600_ ~0.5), unless mentioned otherwise. Cells were harvested by centrifugation at 12,000 × *g* for 10 min at 4 °C.

The bacterial strains and plasmids used in this study are listed in Tables 1 and 2, respectively. *E. coli* strains DH5α and Top10F’ were used for all molecular cloning procedures. The *E. coli* BL21(DE3) and BL21(DE3)omp8 strains were used as hosts for overexpression of BAM and PhoE and purification of OMVs. For overexpression of BAM, consisting of subunits BamA-E, pJH114^[17]^ was used, a kind gift from Harris Bernstein. In this construct, BamE contains an 8xHis tag at its C-terminus for the purification of the assembled BAM complex. Using pJH114 as a basis, we also constructed a BAM complex variant with BamA containing a single cysteine residue in loop 7 of the BamA barrel, inserted between residues Gln753 and Tyr754. The insertion was made using overlap extension polymerase chain reaction (PCR) in three stages. The primers used for the site-directed mutagenesis are listed in Supplementary Table 1. One PCR used the upstream forward primer (pr_M13(-47)_fw) and a reverse primer carrying the cysteine insertion (pr_BamAcys_rv). A second PCR used the corresponding forward primer with the cysteine insertion (pr_BamAcys_fw) and a downstream reverse primer (pr_bamA_Mut_XbaI_rv). The resulting two amplicons were then combined as templates in a third PCR with primers spanning the entire *bamA* gene (pr_M13(-47)_fw and pr_bamA_Mut_XbaI_rv). The resulting amplicon was used to replace *bamA* in pJH114 using restriction enzyme digestion, resulting in pJH114 BamA^cys^, and the successful insertion was confirmed by DNA sequencing (Macrogen).

**Table 1:** List of bacterial strains and their genotype used in this study

**Strains**	**Genotype**	**Reference**
DH5α	*E. coli* K12 *F^-^ φ80lacZΔM15 Δ(lacZYA-argF)U169 recA1 endA1 hsdR17(r_K_^-^, m_K_^+^) phoA supE44 λ^-^thi-1 gyrA96 relA1*	Thermofisher Scientific
Top10F’	*E. coli* K12 *F’{lacIq, Tn10(TetR)} mcrA Δ(mrr-hsdRMS-mcrBC) φ80lacZΔM15 ΔlacX74 recA1 araD139 Δ(ara-leu)7697 galU galK rpsL(StrR) endA1 nupG*	Thermofisher Scientific
BL21(DE3)	*E. coli* B *F^-^ ompT gal dcm lon hsdSB(rB^-^mB^-^) λ(DE3 [lacI lacUV5-T7p07 ind1 sam7 nin5]) [malB^+^]K-12(λS) (DE3)*	Thermofisher Scientific
BL21(DE3)omp8	*BL21(DE3)*, *ΔlamB ompF::Tn5 ΔompA ΔompC*	[16]

**Table 2:** List of plasmids used in this study

**Plasmids**	**Description**	**Reference**
pTRC-His A	IPTG-inducible P*_trc_* *Amp^r^*; Empty vector	Thermofisher Scientific
pJH114	P*_trc_::bamA-bamB-bamC-bamD-bamE(His8), Amp^r^*	[17]
pEH3-PhoE	IPTG-inducible P*_lacUV5_::phoE, Cam^r^*	[18]
pJH114 BamA^cys^	P*_trc_::bamA*^Cys^*-bamB-bamC-bamD-bamE(His8), Amp^r^*	This study

### Isolation of OMVs

OMVs were isolated from bacterial cultures using a differential centrifugation protocol essentially as described^[12]^. Bacterial strains were grown as described above. Cells were then pelleted by low-speed centrifugation at 5,000 × *g* for 10 min at 4 °C, and the supernatant was filtered through a 0.45 µm nitrocellulose membrane to remove residual cells and debris. The filtered supernatant was subjected to ultracentrifugation at 235,000 × *g* for 2 h at 4 °C, and the OMV pellet was resuspended in phosphate-buffered saline (PBS) and stored at -80 °C. The volume was adjusted to the OD_600_ of the bacterial culture it was isolated from to ensure that the OMV yield is approximately normalized for the number of cells harvested.

### Isolation of cell envelopes

Cell envelope (CE) fractions were isolated from bacterial cultures following the protocol described in^[15]^. In short, cell pellets were washed in PBS and then resuspended in a lysis buffer (5 mM Tris-HCl pH 7.4, 100 mM NaCl, 1 mM EDTA) supplemented with a Complete protease inhibitor cocktail (Roche, Germany). Cells were broken by two passages through the One-Shot disruptor (Constant System Ltd., UK) at 1.9 kbar, after which cell debris was removed by centrifugation (10,000 × *g*, 10 min, 4 °C). Soluble protein and membrane fractions were separated by ultracentrifugation (293,000 × *g*, 60 min, 4 °C) and the latter were resuspended in PBS to serve as CE fractions. All steps were performed on ice or at 4 °C and samples were stored at -20 °C until analysis.

### SDS-PAGE and Western blotting

Both cell lysates and OMVs were analyzed by 12% SDS-PAGE (sodium dodecyl sulphate-polyacrylamide gel electrophoresis), Coomassie staining, and Western blotting. Sample loading was corrected based on the OD_600_ of the culture prior to harvesting. As primary antibodies, we used rabbit anti-BAM complex^[19]^, anti-PhoE/OmpC/OmpF (a gift from Jan Tommassen), and anti-FtsH (lab collection). As secondary antibodies, horseradish peroxidase (HRP)-conjugated goat anti-rabbit antibodies (Rockland) were used. Blots were developed using the chemiluminescent ECL Prime substrate (Amersham), and the signal was detected using an Amersham Imager 600 (GE Healthcare).

### Nanoparticle tracking analysis

OMV production was quantified using nanoparticle tracking analysis (NTA) with a NanoSight NS300 instrument (Malvern Panalytical). OMVs were diluted in PBS to achieve an appropriate concentration for analysis (10^7^-10^9^ particles/mL). The size distribution and concentration of OMVs were determined by tracking the Brownian motion of individual particles using NTA software. A standard protocol (3 s × 30 s) with camera level 12, threshold 3, and screen gain 10 was used.

### Heat modifiability assay

Heat modifiability was examined to determine the folding state of β-barrel OMPs. OMV samples were split into two aliquots, of which one was solubilized in 2X sample buffer containing 125 mM Tris-HCl (pH 6.8), 20% glycerol, 10% β-mercaptoethanol, and 0.02% bromophenol blue with 4% SDS and heated at 95 °C for 10 min (denaturing conditions), while the other aliquot was solubilized in semi-native sample buffer (0.4% SDS) and left at room temperature (native conditions). The samples were then subjected to semi-native SDS-PAGE at 4 °C, where the resolving gel lacked SDS, and analyzed by Coomassie Blue staining and Western blotting.

### Pull-down of the bam complex via BamE-8x his tag

BAM-enriched OMVs from BL21(DE3)omp8 harboring pJH114 were solubilized in Buffer A (50 mM sodium phosphate, 500 mM NaCl, 10% glycerol, 10 mM imidazole, pH 7.5) with 2% n-Dodecyl-β-D-Maltoside (DDM) by incubation on ice for 60 min. Insoluble material was removed by ultracentrifugation (200,000 × *g*, 30 min, 4 °C), and the supernatant was diluted in Buffer A containing 0.2% DDM before application to a pre-equilibrated (Buffer A) His SpinTrap^TM^ TALON column (GE Healthcare). The column was incubated with the sample under end-over-end rotation using a Hula mixer at room temperature for 60 min to allow His-tagged BamE to bind. Unbound proteins were collected by centrifugation, followed by three washes with Buffer A + 0.2% DDM to remove non-specifically bound proteins. Proteins were subsequently eluted in two fractions using Buffer B (50 mM sodium phosphate, 500 mM NaCl, 10% glycerol, 400 mM imidazole, pH 7.5) with 0.2% DDM to maintain solubilization of membrane-associated components. Unbound material, wash and elution fractions were collected and analyzed by SDS-PAGE and Western blotting using antibodies against BAM complex to confirm the presence of all its subunits.

### Protease accessibility assay and N-terminal sequencing by edman degradation

The proteinase K (ProtK) assay was essentially carried out as described earlier^[12]^. OMVs were diluted in 50 mM Tris-Cl (pH 7.5), 1 mM CaCl_2_, and subsequently digested at 37 °C for 30 min with 100 μg/mL ProtK (Roche). As a negative control, intact OMVs were incubated under the same conditions in reaction buffer without ProtK. As a positive control, OMVs were solubilized with 1% (v/v) Triton X-100 on ice for 15 min prior to adding ProtK. ProtK reactions were terminated with 0.2 mM phenylmethylsulphonyl fluoride and placed on ice for 10 min. Samples were analyzed by SDS-PAGE, followed by Coomassie staining and Western blotting using antibodies against the BAM complex.

The N-terminal amino acids of the BamA degradation products were blotted onto a polyvinylidene difluoride membrane. The membrane was stained with Coomassie and the bands to be sequenced were excised and sent to Alphalyse, Denmark, for analysis using Edman degradation. The sequentially generated amino acids were identified using high-performance liquid chromatography.

### Surface biotin labeling of BamA extracellular loop region

OMVs were isolated from induced BL21(DE3)omp8 cells harboring pJH114 BamA^cys^ or pJH114 expressing wild-type (WT) BamA as a control. The BamA protein levels in both of these OMV samples were determined using a BSA standard. To specifically label the exposed cysteine, the OMVs were incubated with EZ-Link^TM^ Maleimide-PEG11-Biotin (MPB; Thermo Scientific) at a 1:11 molar ratio of BamA to biotin for 24 h in PBS at 4 °C. A control reaction, lacking the MPB probe, was prepared in parallel using the same conditions. The samples were examined by SDS-PAGE followed by Coomassie blue staining to assess total protein content, and by Western blotting using streptavidin-HRP (Amersham, Cytiva) to detect biotinylated protein.

### Electron microscopy

Transmission electron microscopy (TEM) was essentially performed according to^[20]^. BL21(DE3)omp8 cells transformed with pJH114 and induced for expression of BAM with 100 µM IPTG were collected and pelleted at 5,000 × *g* for 10 min. Cells were washed in PBS and resuspended in 4% paraformaldehyde and 0.5% glutaraldehyde for 2 h at room temperature and subsequently post-fixed with 1% OsO4. The samples were dehydrated in an alcohol series and embedded in epon (LX-112 resin; Ladd Research, Williston, VT, USA). Ultrathin (80 nm) epon sections were collected on Formvar-coated grids. Sections on grids were counterstained with uranyl acetate and lead citrate and imaged using a FEI Tecnai-12 G2 Spirit Biotwin electron microscope (FEI, Eindhoven, the Netherlands).

To visualize OMVs of BL21(DE3)omp8 cultures, samples were directly applied to grids. In this case, the copper grids were first coated with a 5 nm continuous carbon layer and were glow-discharged for 30 s at 25 mAh using a GloQube Plus Glow Discharge System (Electron Microscopy Sciences). OMV samples (4 µL or dilutions thereof) were applied to the grids for 30 s and then blotted off from the side with filter paper. The grid was then washed once with 4 µL of staining solution (2% uranyl acetate), followed by staining for 30 s with the same solution. The stain was blotted off from the side and grids were air-dried before being imaged using a Talos L120C TEM (Thermo Fisher Scientific) equipped with a 4K Ceta CEMOS camera using TIA.

## RESULTS

### Overproduction of the BAM complex in BL21(DE3)omp8 leads to extreme hypervesiculation

**Figure 1:**
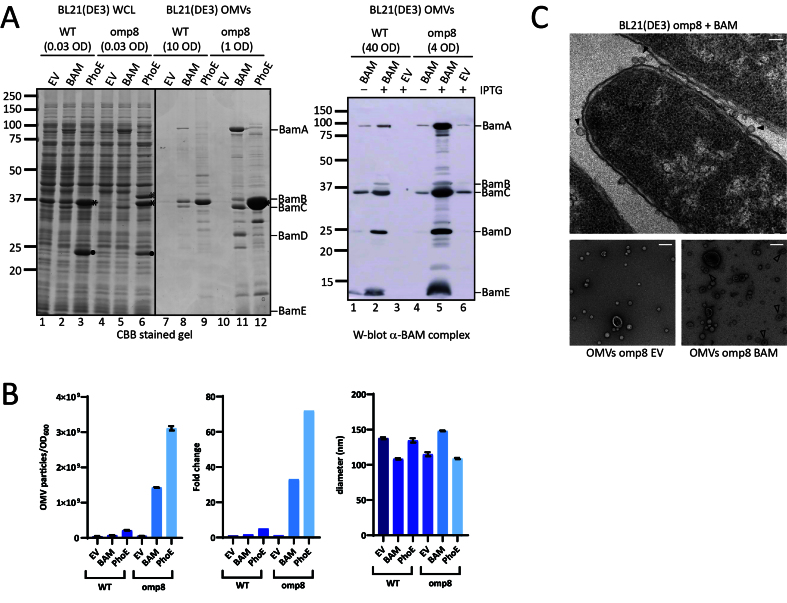
OMV production is increased upon overexpression of the BAM complex and PhoE. (A) CBB-stained SDS-PAGE gel (Left panel) and W-blot incubated with anti-BAM complex antibodies (Right) of WCL and OMVs obtained from cultures of BL21(DE3) WT or BL21(DE3) omp8 (omp8) harboring the empty vector pTRC-His9a (EV), pJH114 expressing the BAM complex (BAM), or pEH3-PhoE expressing PhoE (PhoE). Shown are the relevant parts of the CBB-stained gel, containing samples induced with 100 µM IPTG, and the corresponding regions of the Western blot, containing samples either induced (+) or not induced (-) with 100 µM IPTG. The OD_600_ equivalents of the culture loaded in each sample are indicated. Molecular weight markers are shown on the right, and the positions of BAM subunits are marked on the left based on their expected molecular mass. The black asterisk (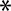) indicates mature PhoE protein; the open asterisk (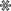) denotes pre-processed PhoE (with signal peptide) that accumulates upon overexpression; and the dot (●) indicates chloramphenicol acetyltransferase encoded by the pEH3-PhoE plasmid; (B) Results of NTA of OMVs isolated from BL21(DE3) WT and omp8 cultures containing EV, BAM, or PhoE. The left panel shows the number of vesicles per OD_600_ of culture, the middle panel depicts the increase in vesicle number relative to WT with EV, and the right panel displays the average vesicle diameter. Bar graphs were generated using GraphPad; (C) Transmission electron microscopy images of BL21(DE3) omp8 cells induced for BAM expression (Top) and OMVs from BL21(DE3) omp8 harboring EV (bottom left) or BAM (bottom right). The scale bar in the top right corner represents 100 nm. Black arrowheads (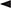) indicate vesicles in the top image, and open arrowheads (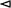) indicate multilamellar OMVs in the bottom left image. OMV: Outer membrane vesicle; BAM: β-barrel assembly machinery; PhoE: outer membrane porin PhoE; CBB: Coomassie brilliant blue; SDS-PAGE: sodium dodecyl sulfate-polyacrylamide gel electrophoresis; WCL: whole cell lysate; EV: empty vector; IPTG: isopropyl β-D-1-thiogalactopyranoside; OD600: optical density at 600 nm; NTA: nanoparticle tracking analysis; WT: wild type; TEM: transmission electron microscopy.

In an attempt to isolate OMVs that are enriched for the BAM complex and have limited interference from other OMPs, we chose to use BL21(DE3)omp8 harboring pJH114 comprising all Bam genes (encoding BamA-E) under IPTG promoter control as the expression strain. The BL21(DE3)omp8 is a genetically engineered derivative of the common BL21(DE3) *E. coli* expression strain and features knockouts of four major OMP genes: *ompA, ompC, ompF,* and *lamB*^[16]^. The strain was made to improve the production of heterologous or homologous recombinant OMPs in the OM by removing the burden of endogenous β-barrel OMPs. WT reference strain BL21 (DE3) and its derivative BL21(DE3)omp8 transformed with pJH114 or the empty expression vector pTRC-His A (EV) as control, were grown and induced with 100 µM IPTG at early log phase. OMVs were purified 2 h after induction started by filtration and ultracentrifugation and analyzed for protein content by SDS-PAGE [Figure 1A]. Whole cell lysates (WCL) were also analyzed to assess overall protein expression. In WCL and OMV fractions of BAM-overexpressing BL21(DE3), the ~88-kDa BamA and ~35-kDa BamC subunits are detected on Coomassie-stained SDS-PAGE gels (Figure 1A, left panel), but the other subunits are also expressed, as is primarily evident from the Western blot analysis (Figure 1A, right panel). Of note, BamB appears diminished compared to BamC-E, which probably reflects either a more loose association or proteolytic degradation, as was observed earlier^[21]^. Strikingly, induced expression of BAM in BL21(DE3)omp8 resulted in large amounts of the BAM subunits in the OMV fraction (Figure 1A, left panel), whereas expression in the cells appeared similar to BL21(DE3) cells. In fact, the OMV fractions had to be diluted 10 times to avoid gel overloading. An explanation for this unexpected result could be that under these expression conditions, vesiculation is strongly increased. To investigate this further, we subjected the OMV fractions to NTA [Figure 1B]. Indeed, BAM overproduction led to a dramatic increase in vesicle production (~30-fold). Of note, analysis of the EV control samples showed that BL21(DE3)omp8 already increased vesicle formation by ~10-fold compared to its parental strain BL21(DE3) without any BAM expression, being 5.5e10^8^ OMV particles/OD_600_ and 4.3e10^7^ OMV particles/OD_600_, respectively, as has been observed before^[22]^. This was attributed to the lack of OmpA, as it forms a connection between the OM and PG that is known to be important for CE integrity^[3]^.

To examine the increased OMV production in more detail, we used transmission electron microscopy (TEM) upon EPON embedding and sectioning to visualize BAM-overproducing BL21(DE3)omp8 cells (Figure 1C, top image). Clearly, many small, round protrusions are visible at the bacterial cell surface, which presumably represent OMVs before they are released into the medium. Purified OMVs from both BAM-overproducing and EV control BL21(DE3)omp8 cells were also analyzed by negative stain TEM (Figure 1C, bottom images). This showed spherical vesicles including some larger, collapsed structures that appear somewhat more abundant in the BAM-enriched sample. Additionally, multilamellar vesicles were observed in the latter sample, but with low frequency. The vesicles appear smaller (20-50 nm) than the size determined by NTA, which is known to be due to fixation and dehydration of the samples.

### Overproduction of a β-barrel OMP in BL21(DE3)omp8 also leads to extreme hypervesiculation

We wondered whether the hypervesiculation was due to overexpression of the BAM complex, or perhaps even reflects a more generic response to the overexpression of any β-barrel OMP. To examine this latter possibility, we overexpressed the unrelated β-barrel OMP PhoE, a trimeric phosphate transporter that is typically only expressed upon phosphate starvation^[23]^. Using again an IPTG-inducible promoter, we detected strong expression of a protein with the expected size of the PhoE monomer in both strains [Figure 1A] that was confirmed to be PhoE by Western blotting using anti-PhoE antiserum (not shown). OMV fractions of PhoE-overproducing BL21(DE3)omp8 clearly showed a massive increase in PhoE content compared to BL21(DE3) WT [Figure 1A] and an even higher concentration of particles by NTA analysis than upon BAM overexpression [Figure 1B]. This strongly suggests excessive vesiculation in the omp8 strain background as a generic response to induced overexpression of β-barrel OMPs.

The OMV fractions appeared to be relatively “clean” upon SDS-PAGE and total protein staining [Figure 1A], arguing against cell fragmentation and the formation of mixed outer-inner membrane vesicles. Indeed, the inner membrane marker protein FtsH was detected exclusively in the WCL samples and not in the OMV fractions [Supplementary Figure 1]. Additionally, the particles in OMV fractions of the hypervesiculation conditions were in the 50-200 nm size range typical of OMVs^[7]^, similar to the control conditions [Figure 1B]. This suggests that the increased number of particles generated by overexpression of β-barrel OMPs in the omp8 strain represent authentic OMVs.

**Figure 2:**
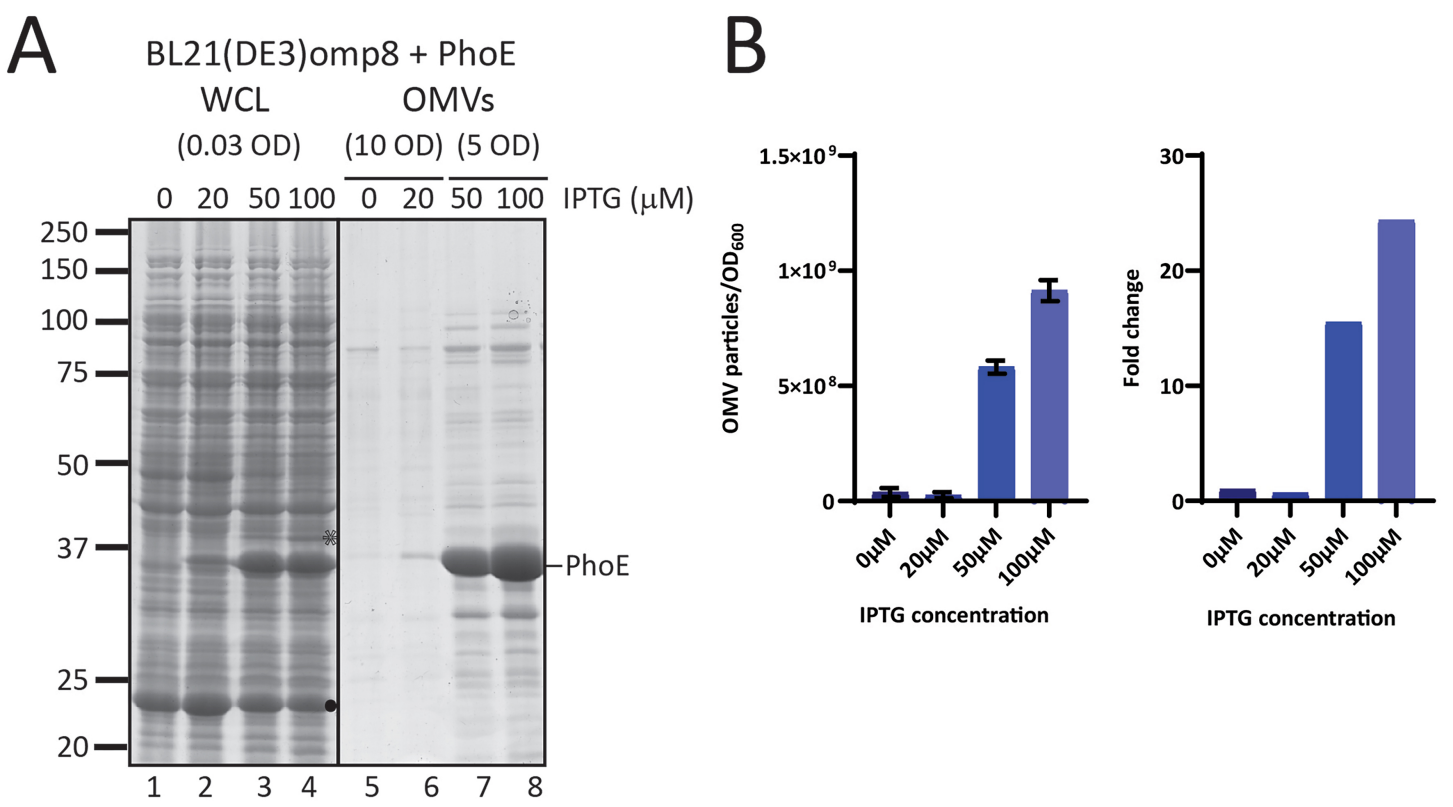
Increased expression of PhoE results in increased production of PhoE-containing OMVs. (A) CBB-stained SDS-PAGE gel showing WCL and OMVs obtained from BL21(DE3) omp8 cultures expressing PhoE from pEH3-PhoE. The gel displays samples from cultures that were not induced (0) or induced with 10, 50, or 100 µM IPTG. The amount of culture loaded, indicated by OD_600_ equivalents, is shown. The position of processed PhoE, based on its expected molecular weight, is marked on the left. The open asterisk (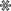) indicates pre-processed PhoE (with signal peptide), while the dot (●) indicates chloramphenicol acetyltransferase encoded by the pEH3-PhoE plasmid; (B) Results of NTA performed on OMVs isolated from BL21(DE3) omp8 cultures expressing different levels of PhoE. The left panel shows the number of vesicles measured per OD_600_ of culture, while the right panel illustrates the fold increase relative to cultures with no IPTG added. Bar graphs were generated using GraphPad. PhoE: Outer membrane porin PhoE; OMV: outer membrane vesicle; CBB: Coomassie brilliant blue; SDS-PAGE: sodium dodecyl sulfate-polyacrylamide gel electrophoresis; WCL: whole cell lysate; IPTG: isopropyl β-D-1-thiogalactopyranoside; OD600: optical density at 600 nm; NTA: nanoparticle tracking analysis.

We noticed that PhoE expression in the omp8 strain harboring the PhoE expression plasmid was higher than the expression of BamA upon BAM complex overexpression [Figure 1A] and this appeared to correlate with a higher production level of OMVs [Figure 1B]. To investigate whether the hypervesiculation is, indeed, proportional to the expression level of a β-barrel OMP, we titrated PhoE expression in the omp8 strain by induction with a range of IPTG concentrations (20-100 µM) to investigate whether the production of OMVs correlates with the extent of β-barrel insertion. Total PhoE expression was clearly titratable up to 100 µM IPTG induction [Figure 2A], showing a parallel increase in OMV production, as evident from the protein staining [Figure 2A] and NTA analysis [Figure 2B] of the OMV fractions. Notably, the increase in OMV production was detected from 50 µM, suggesting that a threshold level of expression may be required to induce OMV formation. Of note, the tested IPTG range of 0-100 µM had no influence on cell growth. These results indicate that hypervesiculation appears to be proportional to the expression of the β-barrel OMP. Overall, they suggest that the absence of major endogenous OMPs in the omp8 strain creates a destabilized OM environment that predisposes the cells to OMV formation, a process that can be amplified by a sudden burst of β-barrel OMP overexpression.

### β-barrel OMPs in BL21(DE3)omp8 OMVs are properly folded

**Figure 3:**
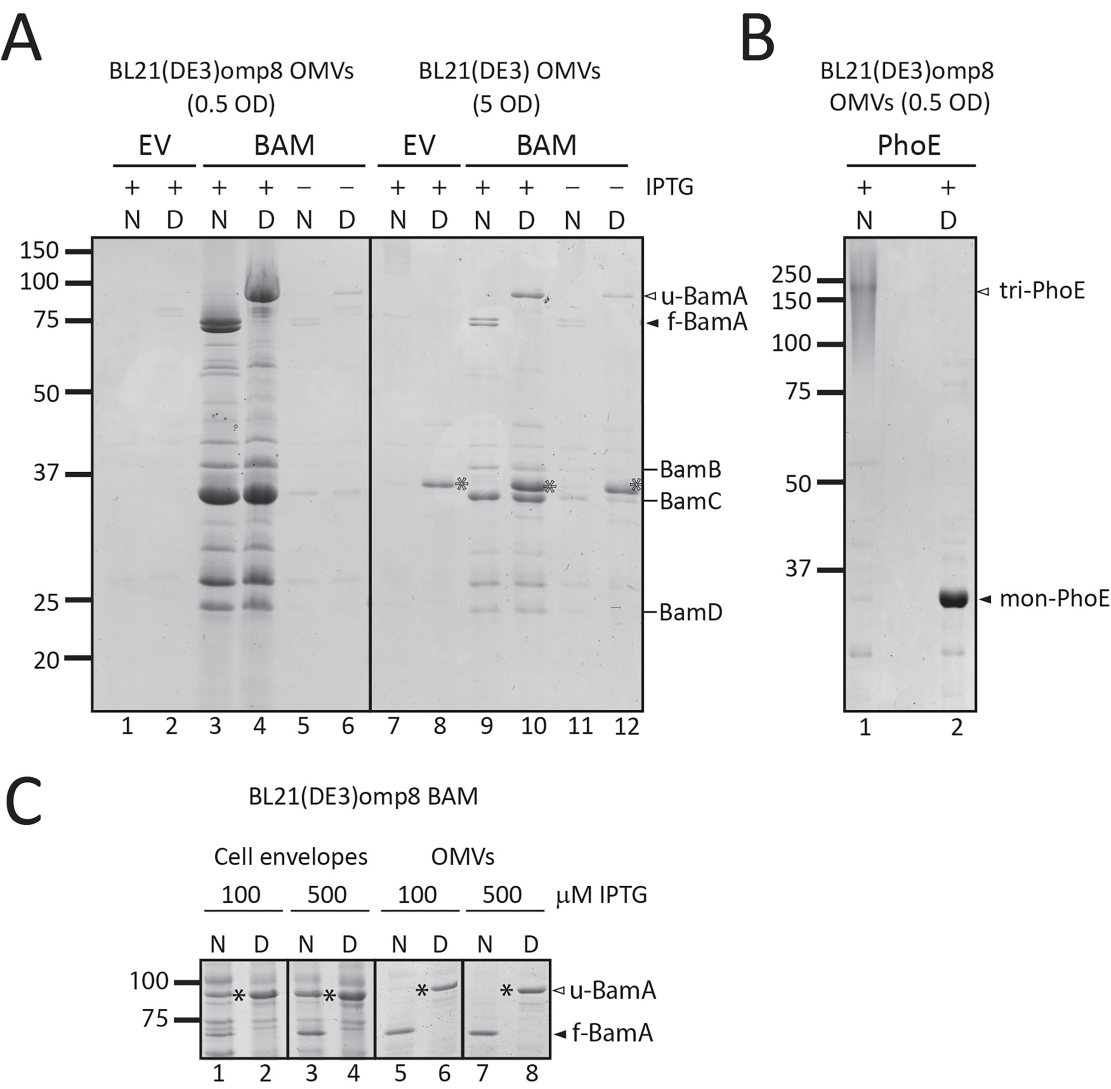
OMVs contain fully folded β-barrel structures of BamA and trimeric PhoE. (A) Relevant parts of CBB-stained semi-native SDS-PAGE gels showing native (N) and denatured (D) samples of OMVs and CEs from BL21(DE3) WT and BL21(DE3) omp8 cells containing either EV or BAM, induced with 100 µM IPTG (+) or not induced (-). The OD_600_ equivalents of the cultures loaded are indicated. Closed and open arrowheads mark the positions of folded (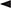) and unfolded (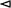) BamA, respectively. The positions of BamB, BamC, and BamD are indicated on the right. The open asterisk denotes the position of unfolded OmpF (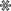) present in the WT samples; (B) Coomassie-stained semi-native gels showing native (N) and denatured (D) samples of OMVs from BL21(DE3) omp8 cells expressing PhoE induced with 100 µM IPTG. The positions of monomeric denatured PhoE (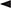
mon-PhoE) and native trimeric PhoE (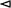
tri-PhoE) are indicated; the trimer runs as a smear due to its association with LPS; (C) Native and denatured samples from CEs and OMVs obtained from BL21(DE3) omp8 cells expressing BAM induced with 100 or 500 µM IPTG. Shown are relevant parts of two gels run in parallel (one for CEs and one for OMVs). The positions of folded (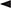) and unfolded (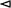) BamA are indicated. The position of unfolded BamA in native samples is indicated by a closed asterisk (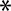). OMV: Outer membrane vesicle; BAM: β-barrel assembly machinery; PhoE: outer membrane porin PhoE; CBB: Coomassie brilliant blue; SDS-PAGE: sodium dodecyl sulfate-polyacrylamide gel electrophoresis; WT: wild type; EV: empty vector; IPTG: isopropyl β-D-1-thiogalactopyranoside; OD600: optical density at 600 nm; OmpF: outer membrane porin F; LPS: lipopolysaccharide; CEs: cell envelopes.

The high level of OMV production in the omp8 strain upon β-barrel OMP expression, combined with the inclusion of the expressed β-barrel OMP in the OMVs, is potentially useful, provided that the OMPs are properly folded. We therefore examined the quality of the OMVs carrying the overexpressed OMPs. First, we analyzed the folding of BamA and PhoE accumulating in the OMVs of the overexpressing strains. In general, the folding state of a β-barrel membrane protein can be assessed using semi-native SDS-PAGE^[24,25]^. β-barrel OMPs in general remain folded even in the presence of low concentrations of SDS, provided that the samples are not heated. This results in an altered, usually faster, migration of the folded protein in the gel compared to a heated, fully denatured sample. Using this “heat modifiability assay” on OMV fractions from the BL21(DE3) and BL21(DE3)omp8 strains, we observed that BamA clearly migrates faster under native conditions irrespective of the host strain used, indicating its folded state [Figure 3A]. This band on SDS-PAGE gels ran under cooled conditions appears as a double band, suggesting different but unknown conformations. In cultures not induced for BAM overexpression, BamA is faintly detectable, but also shifts downward in the native samples. The other visible BAM subunits, BamB, C and D, showed no difference in migration, as expected since these lipoproteins are less stable in the presence of even low concentrations of SDS^[26]^. On the other hand, in the OMVs of the BL21(DE3) strain, we observed heat modifiability of OmpF in both the control EV and Bam-enriched OMVs (Figure 3A, lanes 7-12). Denatured OmpF runs as a single band at ~37 kDa, while native OmpF is a homotrimer that runs higher up in the gel due to its association with LPS^[27]^. We also tested the heat modifiability of overexpressed PhoE in the omp8 strain [Figure 3B] and found that in native OMV samples, all PhoE present appeared to shift to higher positions in the gel, indicative of native PhoE trimers associated with LPS.

Our observation that BamA and PhoE in native samples of BL21(DE3)omp8 OMVs isolated from induced cultures were almost exclusively in a folded conformation was unexpected. OMP overexpression often leads to aberrant folding during translocation across the CE, and one of the functions attributed to OMV shedding is the removal of misfolded OMPs^[28]^. To investigate this further, we compared the folding status of BamA in both OMVs and isolated CEs obtained from BL21(DE3)omp8 cultures induced for BAM expression with 100 and 500 µM of IPTG, the latter concentration inducing full expression of the *bamA-E* operon from pJH114 [Figure 3C]. In contrast to the complete shift of BamA observed in OMVs (Figure 3C, lanes 5-8), the native CE samples still contained considerable levels of BamA migrating at the position corresponding to denatured/unfolded BamA (Figure 3C, lanes 1-4). Apparently, not all BamA expressed and present in the CEs had attained the stable β-barrel conformation, whereas OMVs exclusively contained folded BamA. This effect appeared to be independent of BamB-E, as Omp8 cells that express only BamA also showed exclusion of misfolded BamA from OMVs [Supplementary Figure 2]. Taken together, these data indicate that the overproduced β-barrel proteins in omp8 OMVs are efficiently incorporated and properly folded into their stable β-barrel conformation. Furthermore, nascent or misfolded β-barrel proteins appear to be excluded from these OMVs.

### BL21(DE3)omp8 OMVs are intact and have the correct OMP topology

**Figure 4:**
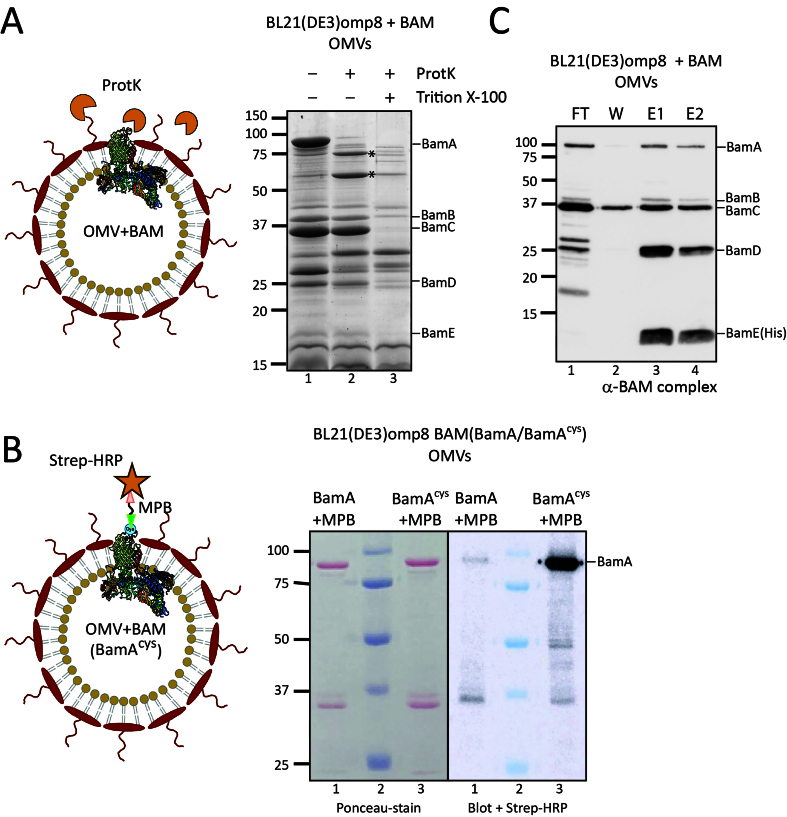
The Bam complex in OMVs is properly assembled, with BamA surface exposed. (A) ProtK treatment of Bam-containing OMVs. The cartoon depicts the rationale of the experiment: OMVs with the BamA subunit of the BAM complex inserted into the OMV membrane, with its extracellular loops exposed. ProtK (orange shapes) is externally added, making these extracellular loops accessible to proteolytic cleavage. The panel on the right shows the relevant part of a CBB-stained gel of OMVs from BL21(DE3) omp8 cells containing BAM, either treated with ProtK or left untreated as a control. The asterisk (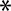) indicates a BamA band resulting from ProtK degradation. Lane 3 contains OMVs treated with Triton X-100 and ProtK, demonstrating the sensitivity of proteins, including BAM subunits, when the OMVs are solubilized. The expected positions of the BAM subunits are indicated on the right; (B) Site-specific labeling of BamA with an engineered cysteine. A cysteine was engineered between Gln753-Tyr754 of BamA, localized in an extracellular loop, allowing labeling on intact OMVs. The cartoon depicts the experiment design: OMVs carrying BAM with the engineered BamA^cys^ are first incubated with a MBP linker that binds the exposed cysteine residue. As a control, OMVs containing BAM with non-mutated BamA are used. The samples are analyzed by SDS-PAGE and Western blotting, with detection using a Strep-HRP. The panel shows the Ponceau stain of the blot (left) and a merged luminescence and bright-light image (right). Lane 2 contains the Precision Plus Protein^TM^ prestained marker (Bio-Rad), which is visible on the right panel due to the merged images. The expected position of BamA is indicated on the right; (C) Pull-down of BAM using His-tagged BamE. The BAM complex encoded by pJH114 includes a His-tagged BamE [BamE(His)], enabling pull-down using TALON resin. The blot shows samples from this pull-down experiment using OMVs solubilized with DDM. The lanes contain the FT, W, and two elution fractions (E1 and E2). The expected positions of the BAM subunits are depicted on the right. OMV: Outer membrane vesicle; BAM: β-barrel assembly machinery; ProtK: proteinase K; CBB: Coomassie brilliant blue; SDS-PAGE: sodium dodecyl sulfate-polyacrylamide gel electrophoresis; Triton X-100: a non-ionic surfactant; MBP: Maleimide-PEG-Biotin; Strep-HRP: Streptavidin-HRP conjugate; DDM: n-dodecyl-β-D-maltoside; FT: flow-through; W: wash; E1/E2: elution fractions 1 and 2.

We next investigated whether the omp8 OMVs produced under hypervesiculation conditions of BAM overexpression are structurally intact and have the expected right-side out topology, similar to the native OM. To this end, the OMVs were treated with ProtK, which is expected to degrade surface-exposed protein domains but is unable to penetrate the membrane of intact OMVs. After ProtK treatment, the samples were analyzed by SDS-PAGE, Coomassie staining, and Western blotting to detect the BAM subunits specifically. The treatment clearly resulted in cleavage of BamA, generating two major fragments of ~74 and ~60 kDa [Figure 4A]. N-terminal Edman sequencing of the first five amino acids of these two degradation products revealed that the fragments both contained the N-terminus of mature BamA (Ala-Glu-Gly-Phe-Val), showing that cleavage has occurred in a C-terminal region, potentially in the exposed loops 6 and 4, respectively. Importantly, the cleavage pattern resembles that generated in whole bacteria under these conditions^[29]^. In contrast, the accessory lipoproteins BamB-E, which are expected to be exposed to the periplasmic side of the OM, appeared protected from ProtK proteolysis [Figure 4A]. It is relevant to note that all BAM subunits were degraded when the OMVs were solubilized with Triton X-100, showing that none of the subunits is intrinsically ProtK-resistant but rather protected by being inside the OMVs.

To further validate the orientation of BamA in the omp8 OMVs, we also used an alternative assay, labeling of a surface-exposed cysteine using a membrane-impermeable probe. For this, we introduced a single cysteine residue into the surface-exposed extracellular loop 7 of BamA^[30]^, between residues Gln753 and Tyr754 (referred to as BamA^cys^, see Figure 4B), and expressed it together with the other BAM subunits using the pJH114 context. This insertion site had been used previously for the introduction of a histidine tag without compromising BAM complex functionality^[30]^. Of note, BamA contains two naturally occurring cysteines at positions 690 and 700 in the partially exposed loop 6, which form a disulfide bond, precluding their use as free labelling sites^[31]^. To confirm that the cysteine insertion does not affect BAM functioning, we complemented depletion of BamA using the *E. coli* JCM166 strain, in which chromosomal *bamA* expression is under control of an arabinose-inducible promoter, forcing cells to rely solely on plasmid-encoded BamA when grown on medium without L-arabinose^[32,33]^. By spotting serial dilutions of JCM166 harboring plasmids encoding the BAM complex with either WT BamA or BamA^cys^ onto plates containing different inducer conditions, we demonstrated that plasmid-based BamA^cys^ expression can complement BamA depletion just like WT BamA, indicating that BamA^cys^ is fully functional.

We then isolated BAM-enriched omp8 OMVs harboring BamA^cys^ or WT BamA as control, and quantified their BamA content using a BSA standard. To assess the accessibility of the engineered cysteine residue, the OMVs, normalized for BamA content, were incubated with MPB [Figure 4B] for covalent labeling of free thiol groups. The samples were then analyzed by SDS-PAGE followed by Ponceau staining, confirming comparable levels of BamA in both OMV samples (Figure 4B, left panel). Subsequent Western blot analysis using streptavidin-HRP demonstrated a distinct chemiluminescence signal at the position of BamA in the BamA^cys^ sample (Figure 4B, right panel). In contrast, WT BamA showed hardly a detectable signal, confirming the absence of reactive cysteines accessible to MBP in the native complex.

Taken together, the data indicate that the OMVs are intact and correctly oriented with a protected lumen corresponding to the periplasm and an exposed OM surface where extracellular BamA loops are accessible.

### Overexpressed BAM subunits assemble into a full complex in BL21(DE3)omp8 OMVs

To confirm that the overexpressed BAM subunits in the OMVs also form a BAM complex, we analyzed complex formation using BamE-His8 for affinity-based purification as shown previously^[17]^. We used the same approach to assess Bam complex formation in the omp8 OMVs that were solubilized with DDM prior to purification on a TALON column. SDS-PAGE followed by Coomassie and Western blot analysis of the different fractions indicated efficient binding of BamE(His) to the column (Figure 4C, FT) and elution of BamE and associated BamA-D in the eluted fractions (Figure 4C, E1 and E2), indicating full BAM complex assembly in the OMVs. The excess amounts of Bam A-D that were detected in the non-bound (FT) fraction probably reflect the superstoichiometric presence of these BAM subunits. Nevertheless, the OMVs appear proficient in BAM complex assembly, and this complex appears to be the main constituent of the omp8 OMVs.

## DISCUSSION

Excessive OMV production has been attributed to loss of OM-PG interactions, accumulation of recombinant proteins in the periplasm, LPS remodeling, and stress invoked by different processes^[4,5,34,35]^. Apart from relieving stress by restoring CE homeostasis, OMVs also have a role in cell-to-cell communication and host-pathogen interactions. Recombinant OMVs are increasingly used in different areas of applied and fundamental research. For instance, they offer a non-live, hence safe, alternative for live attenuated vaccines, providing a facsimile of the OM in an immunogenic particle format^[5,6,9,10]^. Engineered OMVs are also explored for use in cancer immunotherapy and drug delivery^[8,9,10]^. Importantly, they can be used as a structural scaffold for overexpressed OMPs to retain their native conformation and topology in the asymmetric LPS-lipid OM bilayer^[22,36]^. This can be a first step in a purification process or provide an attractive, non-living carrier of suitable size for functional and structural studies of OMPs^[4,5,6]^. The BL21(DE3)omp8 strain lacks the most abundant OMPs and has been shown to be an attractive host for expression of recombinant OMPs that appear abundant in the relatively vacant OMVs^[22,36,37,38]^. We demonstrate here that the omp8 strain has the added advantage that, in response to induced expression of a recombinant β-barrel OMP, it strongly increases the production of OMVs compared to its parental strain BL21(DE3), thus facilitating downstream processing. Of note, the omp8 host already produces ~10-fold more OMVs than the regular BL21(DE3) strain, which is probably due to the lack of OmpA that forms one of the connections to the PG layer underneath the OM^[22]^. However, the hypervesiculation upon induced β-barrel OMP expression is much stronger in the omp8 derivative (~60-fold) and remains unexplained. Of note, we describe here this phenomenon for BL21(DE3) and its omp8 derivative, but have also observed it when overexpressing BAM in *E. coli* K-12 strains. Our results with BL21(DE3)omp8 suggest that other β-barrel proteins may also yield a similar increase in vesiculation in K-12strains, but this remains to be tested.

We were concerned that loss of OM integrity and lysis would contribute to the increase in OMVs produced under these conditions. However, all our analyses of the quality of the OMVs point toward intact, homogeneous OMVs of correct (right-side out) topology, in which the overexpressed OMPs tested (the BAM complex and PhoE) are properly folded and assembled. First, the OMVs produced by omp8 cells when overexpressing β-barrels or not appear to have a similar size distribution, as judged from TEM and NTA particle analysis [Figure 1]. Second, the OMVs appeared impermeable to ProtK, since it only cleaved surface-exposed parts of BamA, suggesting the OMVs are intact and correctly oriented. Third, the β-barrel OMPs that we have overproduced in omp8 cells, BamA and PhoE, appeared to be properly folded as they showed heat modifiability. Similar levels of inserted recombinant OMPs were found by others^[22,23,36,37]^. Interestingly, we found that the BAM OMVs were, in fact, enriched for folded BamA compared to the CEs from which they originated, as the latter contained a considerable amount of unfolded or misfolded BamA. Fourth, overexpression of the BAM subunits resulted in the presence of fully assembled BAM complex in the OMVs, as shown by pull down analysis. It should be mentioned that during the preparation of this study, the Hiller group also reported successful expression and assembly of the BAM complex in omp8 OMVs that were functional and used to develop a high-throughput screening assay for inhibitors of BAM function^[37]^.

All these features of the BL21(DE3)omp8 OMVs indicate that the hypervesiculation observed upon β-barrel OMP overexpression results from an acceleration of the “normal” mechanism of OMV formation, rather than uncovering a radically novel way of vesicle formation. This said, little is known about the molecular mechanism that underlies OMV formation, and a limitation of the current study is that we provide no new insights into this mechanism. Several biogenesis mechanisms have been proposed to explain why certain cues lead to hypervesiculation^[4,5]^. One of the cues is the accumulation of misfolded OMPs in the periplasm, which causes CE stress and a physical outward pressure on the OM. However, our data indicate that unfolded BamA is specifically excluded from the OMVs rather than being recruited and released via OMVs. Additionally, because the omp8 strain lacks most natural OMPs, there should be sufficient capacity of the machinery involved in the trafficking, insertion, and folding of OMPs, as shown by the abundance of OMPs in omp8-derived vesicles^[22,23,36,37]^. Obviously, the omp8 OM composition is completely different from the normal OM and this could impact OMV biogenesis when a sudden burst of β-barrel OMP biogenesis must be accommodated. The lack of OmpA weakens the integrity of the OM by reducing stabilizing connections with the PG, which on its own leads to moderate hypervesiculation^[39]^. The lack of the porin OmpC may affect lipid homeostasis in the OM as it is connected to the Mla system. This machinery is involved in the retrograde transport of phospholipids that have aberrantly entered the outer LPS leaflet^[40]^. The imbalance between OMPs and LPS may lead to this accumulation of phospholipids, a condition that is known to cause vesiculation^[41]^. Importantly, in addition to the consequences of the lack of individual OMPs, the whole fabric of the OM is different in the omp8 strain. AFM measurements have shown that OMPs are normally packed in a mosaic of ordered networks, held together by LPS molecules. These rather immobile protein islands with high OMP density cover ~70% of the cell surface^[42,43]^. They also contain BAM complexes that may operate in “precincts” within these islands, where a few complexes are assembled through interactions between BamA and BamB of neighboring complexes^[44]^. It is possible that, in the absence of this supramolecular context in the OM of the omp8 strain, its BAM complexes will insert and fold overexpressed β-barrel OMPs, after which they may assemble in irregular oligomeric structures. These, in turn, may change the local curvature of an OM whose integrity is already affected by the lack of load-bearing OMPs, triggering OMV formation. Alternatively, the sudden change in OMP-LPS balance may induce OMV formation via stress-related signaling pathways, as multiple CE stress systems monitor OM integrity^[41]^.

It will be interesting to further investigate the mechanism of the extreme vesiculation in the omp8 strain under OMP overexpressing conditions, as it may also provide insight into the natural mechanism of shedding of OMVs in WT *E. coli* cells. However, irrespective of the mechanism, the generation of high levels of robust omp8 OMVs that contain densely packed and well-folded recombinant OMPs may prove a valuable tool in future structural and functional studies. Given their size and homogeneous composition, the OMVs appear particularly suited for Cryo-EM and Cryo-ET studies of any overexpressed recombinant OMPs.

## DECLARATIONS

### Availability of data and materials

Data and materials are available from the corresponding author upon reasonable request.

### Copyright

© The Author(s) 2025.
