# Supplementary material for: Overproduction of β-barrel outer membrane proteins in Escherichia coli BL21(DE3) induces hypervesiculation
Source: Extracell Vesicles Circ Nucl Acids. 2025 Jul 17;6(3):386–402. doi: 10.20517/evcna.2025.27 (PMC12540271; doi:10.20517/evcna.2025.27)
Supplement: Supplementary file 1 [file evcna-6-3-386-SupplementaryMaterials.pdf]

Supplementary Materials

Overproduction of β-barrel outer membrane proteins in *Escherichia coli* BL21(DE3) induces hypervesiculation

Saloni Sahu[1], Gregory Koningstein[1], Catalin M. Bunduc[1], Nicole van der Wel[2], Joen Luirink[1], Peter van Ulsen[1]

[1]Molecular Microbiology, AIMMS and A-LIFE, Vrije Universiteit Amsterdam, De Boelelaan 1108, Amsterdam 1081 HZ, The Netherlands.
[2]Electron Microscopy Centre Amsterdam, Amsterdam University Medical Centre, Amsterdam 1100 DE, The Netherlands.

Correspondence to: Dr. Peter van Ulsen, Molecular Microbiology, AIMMS and A-LIFE, Vrije Universiteit Amsterdam, De Boelelaan 1108, Amsterdam 1081 HZ, The Netherlands. E-mail: j.p.van.ulsen@vu.nl

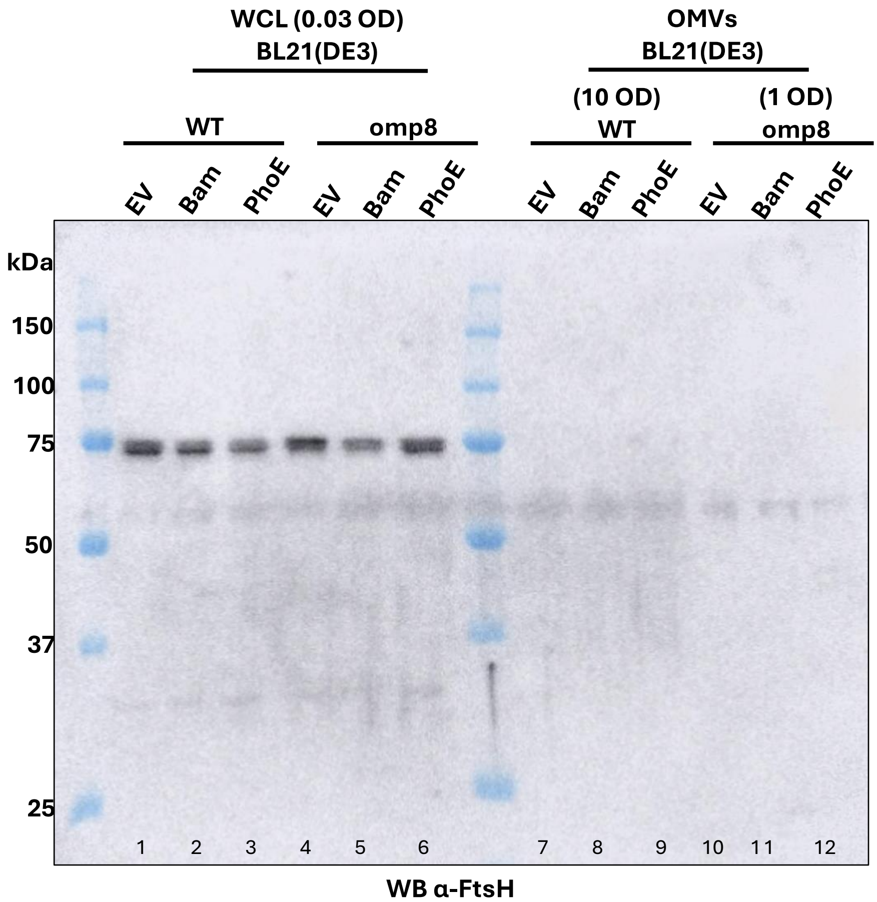

Supplementary Figure 1. OMVs of BL21(DE3) and BL21(DE3)omp8 do not contain the inner membrane marker protein FtsH. Shown is the merged bright-light and chemiluminescence image of a blot incubated with anti-FtsH antiserum. On the left WCL samples are loaded (lanes 1-6), on the right OMV samples isolated from the same cultures. Of note, the same samples were used for Figure 1. of the same cells as used for Figure 1. Note that there is only a signal for FtsH in the WCL samples, indicating that the OMVs are devoid of the inner membrane marker protein FtsH.

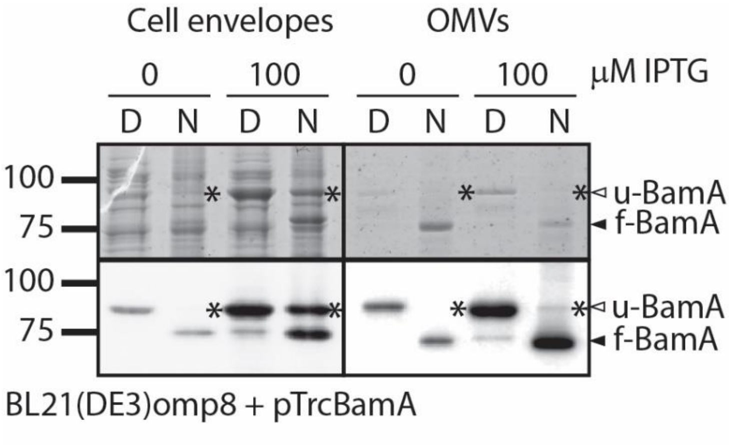

Supplementary Figure 2. Unfolded BamA accumulates in Cell Envelopes, not in OMVs. Coomassie-stained gel and Western blot of native (N) and denatured (D) samples of cell envelopes (CEs) and OMVs obtained from BL21(DE3)omp8 cultures induced for

expression of BamA from plasmid pTRC-BamA with 100 µM IPTG and their non-induced controls. Blots were incubated with anti-BamA antiserum. Shown are the relevant parts of two gels and blots (one each for the CEs and the OMVs). Indicated are the running positions of unfolded and folded BamA. The position of the unfolded BamA in native samples is indicated by "*". The plasmid pTRC-BamA is a derivative of pJH114, but lacks the *bamB-bamE* genes and only encodes BamA. BamA on its own also induced formation of OMVs. Clearly, and similar to what was found for the BAM complex, all the BamA in OMVs shifts to a lower position in the gel indicative of its fully folded status in OMVs, whereas part of the expressed BamA in CEs remains at the non-folded position in native samples of cell envelopes, indicative of being not folded.

Supplementary Table 1. Primers used for insertion of cysteine between Gln753-Tyr754 in BamA

Primers	Description
pr_BamAcys_fw	GGGATTCCAGCCAAT<u>**GCT**</u>ATTCTGGATATCCGGACT
pr_BamAcys_rv	GATATCCAGAATA<u>**GCA**</u>TTGGCTGGAATCCCAGTTTGTATCCCA
pr_M13(-47)_fw	CGCCAGGGTTTTCCCAGTCACGA
pr_BamA_Mut_XbaI_rv	GCTCTAGACCTGGCAGCAGTAATTTACGCAATT

The cysteine insertion is underlined in the respective forward and reverse primers.
